# Endoscopic Management of the Difficult Bile Duct Stones: A Single Tertiary Center Experience

**DOI:** 10.1155/2016/8749583

**Published:** 2016-11-24

**Authors:** Bülent Ödemiş, Ufuk Barış Kuzu, Erkin Öztaş, Fatih Saygılı, Nuretdin Suna, Orhan Coskun, Adem Aksoy, Zeliha Sırtaş, Derya Arı, Yener Akpınar

**Affiliations:** Department of Gastroenterology, Turkiye Yuksek Ihtisas Education and Research Hospital, Ankara, Turkey

## Abstract

*Background*. Most common bile duct (CBD) stones can be removed with standard techniques using endoscopic retrograde cholangiopancreatography (ERCP), but in some cases additional methods are needed. In this study we aimed to investigate the management of patients with difficult stones and the factors that affect the outcome of patients that have undergone periodic endobiliary stenting.* Materials and Methods*. Data of 1529 patients with naive papilla who had undergone ERCP with an indication of CBD stones was evaluated retrospectively. Stones that could not be removed with standard techniques were defined as “difficult stones.” Cholangiograms of patients who had difficult stones were revised prospectively.* Results*. Two hundred and eight patients (13.6%) had difficult stones; 150 of these patients were followed up with periodic endobiliary stenting and successful biliary clearance was achieved in 85.3% of them. Both CBD (*p* < 0.001) and largest stone size (*p* < 0.001) were observed to be significantly reduced between the first and the last procedure. This difference was even more significant in successfully treated patients.* Conclusions*. Periodic endobiliary stenting can be used as an effective treatment for patients with difficult stones. Sizes of the CBD and of the largest stone are independent risk factors that affect the success rate.

## 1. Introduction

Common bile duct (CBD) stones are the most common indication for endoscopic retrograde cholangiopancreatography (ERCP) in clinical practice. Most CBD stones can be removed by extraction with balloon and/or dormia basket following endoscopic sphincterotomy, and clearance of the biliary system is achieved [1]. However, in approximately 15% of patients with CBD stones clearance of the biliary system cannot be obtained using these standard techniques and these kinds of stones are termed as “difficult stones.” The properties of difficult stones are stone diameter > 1.5 cm, number of stones > 3, existence of periampullary diverticula, impaction of the stone, and narrowing of the biliary duct distal to the stone [1–3]. Additional interventional techniques such as electrohydraulic/laser lithotripsy, which is not widely available in most centers, or mechanical lithotripsy can be used for difficult stones in the same session when standard methods have failed [3, 4].

Endobiliary stent placement in order to achieve biliary decompression or extracorporeal shock wave lithotripsy (ESWL) following nasobiliary drainage can be used when all interventions fail. Endobiliary stenting provides further time for deciding the consequent follow-up and where it also helps to reduce the diameter of stones leading to improved success rates with standard techniques to remove stones in the following ERCP sessions [5]. Despite all available techniques, it is sometimes impossible to remove the difficult stone and these patients are referred for surgery as a last treatment option.

The primary aim of this study was to assess the general characteristics of patients with difficult stone who had undergone ERCP for CBD stones. The secondary aim was to investigate the efficacy of periodic endobiliary stenting for difficult stones and the factors that affect the success rates of this procedure.

## 2. Materials and Methods

### 2.1. Study Design and Patients

This study was carried out in ERCP unit of Türkiye Yüksek İhtisas Hospital. Data of patients with naive papilla who had a diagnosis of CBD stones proven by radiological or endosonographic imaging were enrolled in the study, who were extracted from 11.785 patients who had undergone ERCP between January 2008 and June 2013. Patients with a history of sphincterotomy, Mirizzi syndrome, intrahepatic biliary stones, need for percutaneous transhepatic cholangiography, pancreatobiliary malignancy, or multiorgan dysfunction due to suppurative cholangitis were excluded.

### 2.2. ERCP Procedure and Stone Extraction Techniques

**Figure 1:**
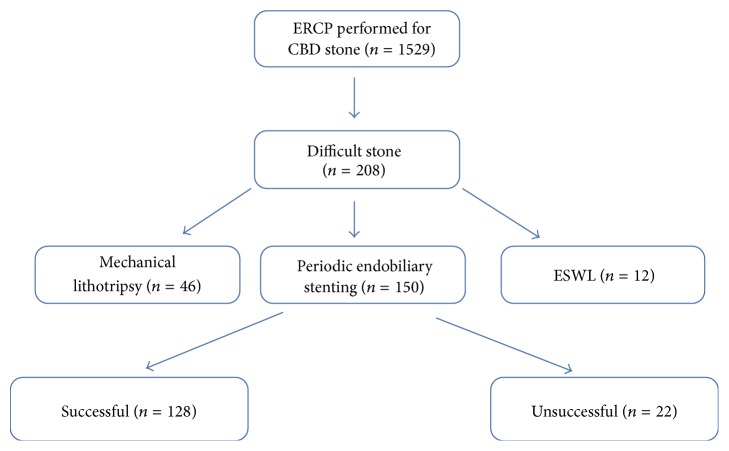
Design of the study population (ESWL: extracorporeal shock wave lithotripsy).

All procedures were performed by three experienced pancreaticobiliary endoscopists using an Olympus video duodenoscope (Olympus TJF 260 or JF 260, Tokyo, Japan). Every endoscopist had experience with ERCP for more than 3 years, and all of them were individually performing more than 700 ERCP procedures per year. Also all of them had more than 90% technical success rates. The ERCP procedure is performed in our clinic as follows: the CBD is cannulated selectively either directly or with fistulotomy and a guidewire is placed into the CBD. Sphincterotomy is then performed using an endoscopic sphincterotome placed using a guidewire. Existing stones in the biliary system are extracted with an extraction balloon or dormia basket after sphincterotomy. In case of any stricture distal to the stone, balloon dilation with 6 or 8 mm balloons is performed before extraction. If transpapillary dilatation is required, sphincteroplasty with a 12 mm balloon is performed. Biliary clearance is then confirmed by injecting radio-contrast into the CBD via balloon catheter and the balloon is then pulled out from the CBD into the duodenum. Stones that cannot be extracted in the first ERCP procedure using this technique are defined as difficult stones. In order to further define this with details, if the stone cannot be extracted despite one or more of the methods above, increasing complication risk due to recurrent extraction attempts is realised (e.g., bleeding in the incision site due to recurrent trauma, recurrent cannulation of the pancreatic duct with balloon or basket, overextension of the CBD with the trauma of the Dormia basket, and increasing risk of perforation), prolonged procedure time that negatively affects the attention of the ERCP team, which is consequently increasing the risk of complications, prolonged sedation time that can bring anaesthesia induced complications, and finally the decision of the experienced endoscopist that the extraction is not possible; these stones are defined as difficult stones. In our clinic, management of the difficult stones is planned in four ways: (1) Mechanical lithotripter is used to break the stone into smaller parts; (2) sequential ERCP procedures within 2-3-month periods are performed after placing one or multiple endobiliary stents (Amsterdam type or double pigtail plastic stents); (3) ESWL is performed following nasobiliary drainage marking; (4) surgical removal of the stones is performed. Design of the study is shown in Figure 1.

Management of the difficult stones was performed according to these consequent criteria: If the procedure time is not so prolonged and anaesthesia induced complication risk was not elevated, mechanical lithotripsy was used with the decision of the endoscopist. If mechanical lithotripsy succeeded, the procedure was finished during the same procedure. In case of failure with mechanical lithotripsy (due to inability to hold the stone with a basket or large diameter of the stone exceeding the basket size) periodic stenting or ESWL decision was made with the ERCP according to criteria such as patient compliance to stenting periods, difficulty in recurrent procedures (due to serious comorbidity), socioeconomic aspects of the patient, and available facilities of the hospital at the time of the procedure. Finally surgery was suggested in case of failure with all the mentioned three methods and according to the patient's choice.

### 2.3. Study Data

Demographic features, results of the imaging studies, and clinical data were noted on a single form for each case. This form contained the following data: (1) endoscopic and radiological findings of the patients; CBD width, existence of periampullary diverticula, existence of biliary opening anomaly, impaction of the stone, and biliary stricture distal to the stone; (2) management plan of cases after inability of biliary clearance with standard techniques (mechanical lithotripsy, periodic endobiliary stenting, ESWL, and surgery); (3) medical history; gastric surgery (Billroth I, Billroth II, and simple gastroenterostomy) and cholecystectomy; (4) complications of ERCP (post-ERCP pancreatitis, bleeding, cholangitis, and perforation). (5) Serum biochemical parameters studied in our center before the ERCP procedure: amylase, total bilirubin (Tbil), aspartate aminotransferase (AST), alanine aminotransferase (ALT), alkaline phosphatase (ALP), gamma glutamyl transferase (GGT), glucose, and international normalized ratio (INR).

Additionally images of the patients with periodic endobiliary stenting due to difficult stone were prospectively reevaluated. All cholangiographic images for all sessions were revised and CBD diameters at the first and last session, number of the stones, and diameter of the largest stone and its change were also noted using the diameter of the duodenoscope as a reference.

The data of the patients were collected from the AviCenna Medical Data Managing System (Dataselin formation systems, Ankara, Turkey). AviCenna Medical Data Managing System supports internationally approved standard data, such as ICD-10, SNOMED, ATC, and GMDN.

### 2.4. Statistical Analysis

Statistical analysis of the data was performed with Statistical Package for Social Sciences (SPSS) version 18 (SPSS Inc., Chicago, IL, United States) package software. Continuous variables were expressed as mean (± standard deviation), and median (interquartile range: 25–75 percentiles) when available, and categorical variables were expressed as number and percentage (*n*, %). Student's *t*-test was used to compare the groups with continuous variables while Pearson chi-square test and Fischer exact chi-square test were used to compare groups with categorical variables. Probable factors that were defined in previous analysis were used in multivariate analysis to predict the existence of difficult stones and the success rate of periodic endobiliary stenting using logistic regression analysis of independent predictors. Hosmer-Lemeshow test was used for compatibility of the model. Results with *p* values less than 0.05 were defined to be statistically significant.

## 3. Results

Total of 1529 patients were enrolled in the study. The mean age was 63.3 ± 17 years and 61.9% of the patients (*n* = 947) were female. Biliary clearance was accomplished in 86.4% of the patients (*n* = 1321) in the first ERCP procedure using standard techniques, while the percentage of patients with difficult stones was observed to be 13.6% (*n* = 208) (Table 1). The mean age was higher in patients with difficult stones when compared to the easy group (66 years versus 62.9 years; *p* = 0.015). In terms of laboratory parameters, patients with difficult stones had lower enzyme levels, except for ALP; however bilirubin levels were significantly higher than in the easy group. History of cholecystectomy (41.9% versus 25.6%; *p* < 0.001), biliary opening anomaly (7% versus 2%; *p* < 0.05), stricture distal to the stone (13.6% versus 0.22%; *p* < 0.001), impaction of the stone (8.6% versus 0.15%; *p* < 0.001), and dilation of the CBD (94.2% versus 70.4%; *p* < 0.001) were significantly higher in the difficult stone group. Existence of periampullary diverticula and gastrointestinal surgery were not individually observed to be a risk factor for difficult stone (*p* > 0.05). Additionally, the total complication rate including cholangitis, pancreatitis, and bleeding was observed to be higher in the difficult stone group; however this did not reach statistical significance (8.1% versus 5.6%; *p* > 0.05).

Forty-six patients (22.1%) with difficult stones underwent mechanical lithotripsy in the first session when standard techniques failed to achieve biliary clearance. ESWL was performed in 12 of 162 remaining patients and 8 of them successfully achieved biliary clearance. A total of 150 remaining patients were scheduled for periodic endobiliary stenting. In these patients who were examined after periodic endobiliary stenting, CBD diameters during the initial session were determined to be significantly higher when compared with the last procedure (15.68 ± 4.96 mm and 14.60 ± 5.48 mm, resp., *p* < 0.001). Similarly, the diameter of the largest stone was observed to decrease significantly after periodic stenting (17.41 ± 7.44 mm versus 15.85 ± 7.73 mm; *p* < 0.001).

When 150 patients that underwent periodic endobiliary stenting were divided into two groups in terms of successful biliary clearance (successful group; 85.3%, *n* = 128, and unsuccessful group; 14.7%, *n* = 22), there was no difference in terms of gender, age, total number of ERCP sessions, follow-up time, use of additional techniques (balloon sphincteroplasty and mechanical lithotripsy), and initial laboratory findings before the first session (*p* > 0.05) (Table 2). Similarly, there was no difference in the successful group and unsuccessful group by means of history of cholecystectomy (36.7% versus 39.2%, respectively; *p* = 0.473), existence of periampullary diverticula (14.8% versus 4.5%, respectively; *p* = 0.308), history of gastric surgery (3.9% versus 0%, respectively; *p* = 0.446), impaction of the stone (10.9% versus 13.6%, respectively; *p* = 0.375), and stricture distal to the stone (21% versus 27.2%, respectively; *p* = 0.379). However, the CBD diameter at the first ERCP session (14.8 mm versus 20.7 mm, respectively; *p* = 0.006) and diameter of the largest stone (15.8 mm versus 27.7 mm, respectively; *p* < 0.001) were observed to be significantly lower in the successful group. Similarly, CBD diameter in the last session (13.1 mm versus 19.3 mm; *p* < 0.001) and diameter of the largest stone (13.3 mm versus 26.1 mm; *p* < 0.001) were significantly lower in the successful group. From another point of view, after periodic endobiliary stenting, the CBD diameter was observed to decrease by 82.8% of the patients in the successful group whereas it was only 31.8% in the other group (*p* = 0.001). However, a decreased diameter of the largest stone did not show a significant difference between these groups (75.7% versus 57.1%, respectively; *p* = 0.413). According to multivariate analysis with logistic regression, only CBD diameter (odds ratio (OR): 0.780 (95% CI: 0.681–0.893; *p* = 0.001)) and the diameter of the largest stone (OR: 0.808 (95% CI: 0.726–0.898; *p* = 0.001)) were defined to be independent risk factors that affected success (Table 3).

When we evaluated the success rate according to ERCP session in the successful group (*n* = 128), biliary clearance which was achieved at the second ERCP session after stenting was 40.6% (*n* = 52), whereas it was 32.8% (*n* = 42) at the third session and 26.6% (*n* = 34) at more than three [4–9] ERCP sessions.

When complications are considered, three patients had cholangitis due to stent occlusion, two patients had bleeding, and three patients had pancreatitis in the successful group. On the other hand, in the unsuccessful group, two patients had bleeding, one patient had cholangitis due to stent occlusion, one patient had pancreatitis, and one patient had perforation due to stent migration. Cholangitis as a late complication due to stent occlusion was observed in only one patient within the first month after stenting whereas the other three occlusion induced cholangitis cases were observed between 2 and 5 months.

## 4. Discussion

Management strategies have been evolving in recent years as research on the biliary system has been growing and developing. Particularly the increasing availability of diagnostic tools such as magnetic resonance cholangiopancreatography and endoscopic ultrasound has influenced the diagnostic accuracy and awareness of CBD stones. Additionally laparoscopic cholecystectomy is preferred against open surgery and this approach has caused ERCP to be the initial step for management of CBD stones [3, 4]. According to recent knowledge our study has the largest number of patients in therapeutic approach for CBD stones. Among this large number of patients CBD stones were successfully removed with standard techniques at the first ERCP session in 86.4% of them. Recent literature has reported that difficult stones can be seen in 7–20% of patients while in our study we have similarly experienced difficult stones in 13.6% of patients, and additional interventions were indicated [3, 6–9]. We have observed that general features of the patients with difficult stones are similar to the recent literature, such as older age, dilation of the CBD, impaction of the stone, stricture distal to the stone, and opening anomaly of the papilla [1, 6, 9]. Additionally, it must be noted that patients with difficult stones had higher bilirubin levels, while they had relatively lower hepatic enzyme levels. However, existence of periampullary diverticula did not show any association with difficult stones. There are reports that support our findings while there are others that suggest the opposite [2, 6, 7, 9]. Probably these diversions are due to differences in ERCP experience of the reporting centers.

Periodic endobiliary stenting is being used as an alternative treatment approach in order to induce biliary drainage for patients that have difficult stones and cannot tolerate advanced endoscopic interventions and/or surgery [10]. Additionally, it has been recently suggested that this approach can reduce the diameter or even totally clear off the CBD stone and so can be a bridging or an adjuvant therapy for definitive treatment. This has been suggested to be the result of the mechanical irritation induced by the friction force between the stone and the stent, so that the stone is fragmented into smaller pieces and the diameter is reduced. The success rate after periodic endobiliary stenting is reported to be between 62.5 and 95% in recently published studies [7, 8, 11]. In our study we have achieved biliary clearance in 85.3% of patients with difficult stones using periodic endobiliary stenting. We have observed a significant decrease in the diameters of both CBD and the largest stone after periodic endobiliary stenting for difficult stones. The decreases in diameters of the CBD and the largest stone were more prominent in the successful group when compared to the unsuccessful group and they were the only significant difference between these two groups. The diameters of the CBD and the largest stone were found to be independent risk factors that affect the success of biliary clearance.

The mean number of ERCP sessions among patients that underwent periodic endobiliary stenting was three and the follow-up period was long. These factors can explain the high success rate. As a finding that supports this theory, the success rate of a single ERCP session after endobiliary stenting was reported to be 60%, while this rate was observed to increase up to 90% in patients with periodic endobiliary stenting within a longer follow-up period [5, 7, 8]. In our study we have observed that biliary clearance was successfully achieved after more than three sessions in most of the patients. However, complications such as cholangitis and pancreatitis can be encountered after periodic endobiliary stenting. In our study we have observed cholangitis and pancreatitis in 4 patients (2.6%) for each and as a rare complication, duodenal perforation occurred due to stent migration. There was no complication related mortality in our study. There are publications that report low complication rates similar to our study; however there are also up to 60% complication rates in the literature [7, 8, 12]. Probably these different rates are due to the changes in intervals of control and stent substitutions. There is still no exactly defined time for stent substitution and control; however it is also known that complication rates increase as the interval of stent substitution is longer [13]. Thus, according to recent reports, the mean period of substitution interval changes between 2 and 3 months and with this planning complication rates are decreasing [7, 8, 11].

Additional techniques such as mechanical lithotripsy (ML) and balloon dilation of papilla can be used during the same ERCP procedure for patients with difficult stones. Mechanical lithotripsy has been first defined in 1982 and recently it is almost a standard treatment modality for the patients with difficult stone [14]. The aim in mechanical lithotripsy is to cover the stone which was captured with Dormia basket inside choledocus with a polytetrafluoroethylene sheath and destruction of the stone by applying an increasing force with the help of the rotation of the metal part [3]. ML has been reported to be successful to induce biliary clearance by 80% in the literature [15–18]. Balloon dilation for papilla following endoscopic sphincterotomy has been first defined in 2004 [19]. Studies following this approach have shown that the technique was highly effective among patients with difficult stones [20–23]. However when it was compared with sphincterotomy only, there are conflicting results about it on increasing post-ERCP pancreatitis [24]. Additionally when it is compared with sphincterotomy only, it has been shown to reduce the need for mechanical lithotripsy among the patients with difficult stone [20].

Another treatment option for difficult stones is ESWL. In this technique shock waves produced by an electromagnetic membrane are focused on the stone in order to break it [25]. As most of the biliary stones are radiolucent, nasobiliary drain catheters have to be placed and the stone is marked with radio-contrast before the ESWL procedure, which is directed under fluoroscopy [4, 25]. In our study the success rate of ESWL was found to be 66.6% which is similar to recent knowledge where it is reported to be between 53% and 90% [3]. However this method is recently leaving its place to a more efficient technique, which is cholangioscopy guided laser lithotripsy [4]. Laser lithotripsy is application of laser fibers on the stone which is visualised with cholangioscope and divides the stone into its fragments [3, 26]. The main disadvantage of it is its rare availability in some centers when compared to other techniques; however it is becoming a preferred technique and it has been shown to be highly effective in achieving biliary clearance [27–30]. Outcome of additional techniques for the patients with difficult stone has been shown in Table 4.

In conclusion, ERCP is a highly effective method in treatment of CBD stones. According to our study, 13.6% of the patients with CBD stones had difficult stones. Periodic endobiliary stenting significantly reduced the diameter of the CBD and the largest stone and this effect was more prominent among successfully treated patients. Additionally, the diameters of the CBD and of the largest stone were found to be independent risk factors that affected success rates.

## Figures and Tables

**Table 1:** Main differences between patients with easy and difficult stones.

Variable	Easy stone (*n*: 1321)	Difficult stone (*n*: 208)	*p* value
Age (year)	62.9 ± 17	66 ± 16.9	0.015
Gender: women/men; *n* (%)	811 (61.4)/510 (38.6)	136 (65.4)/72 (34.6)	0.267
Laboratory findings			
Glucose (mg/dL)	115 ± 45.1	115.6 ± 60.3	0.915
ALT (U/L)	80 (5−2665)	48 (1−643)	<0.001
AST (U/L)	59 (2−1851)	47 (10−607)	0.004
GGT (U/L)	278 (6−2400)	215 (4−5811)	0.016
ALP(U/L)	183 (9−2069)	200 (16−2105)	0.418
Amylase (mg/dL)	208 ± 551	114.4 ± 158.9	0.954
Total bilirubin (mg/dL)	5.3 ± 4.58	9.6 ± 11.8	0.006
Endoscopic/radiological findings; *n* (%)			
Cholecystectomized patients	318 (%25.6)	78 (41.9)	<0.001
Papilla in bulbus	16 (1.2)	7 (3.5)	0.025
Papilla in 3rd portion	11 (0.8)	7 (3.5)	0.006
Periampullary diverticula	236 (17.9)	29 (13.9)	0.063
History of gastric operation	14 (1.1)	4 (1.9)	0.100
Impacted stone	2 (0.15)	18 (8.6)	<0.001
Stricture distal to the stone	3 (0.22)	40 (13.6)	<0.001
Number of patients with dilated CBD; *n* (%)	743 (70.4)	114 (94.2)	<0.001

Values are presented as number (%), mean ± SD, or median (range).

ALT: alanine aminotransferase; AST: aspartate aminotransferase; CBD: common bile duct; GGT: gamma glutamyl transferase; ALP: alkaline phosphatase.

**Table 2:** Comparison of groups that underwent periodic endobiliary stenting.

Variable	Successful (*n* = 128)	Unsuccessful (*n* = 22)	*p* value
Age (year)	67.8 ± 17.4	61 ± 15.2	0.052
Gender: women/men; *n* (%)	99 (77.3)/29 (22.7)	19 (86.4)/3 (13.6)	0.340
Glucose (mg/dL)	114 ± 34.2	111 ± 37.9	0.314
Laboratory findings			
ALT (U/L)	72.9 ± 70.7	53.4 ± 62.4	0.082
AST (U/L)	70.9 ± 76.5	48.7 ± 36.3	0.416
GGT (U/L)	334 ± 568	265 ± 329	0.329
ALP (U/L)	276 ± 289	364 ± 326	0.297
Amylase (mg/dL)	128 ± 187	82 ± 41	0.888
Total bilirubin (mg/dL)	3.47 ± 6.8	2.7 ± 4.8	0.289
Endoscopic/radiological findings *n* (%)			
Cholecystectomized patients	47 (36.7)	11 (39.2)	0.473
Papilla in bulbus	7 (5.5)	0	0.559
Papilla in 3rd portion	4 (3.1)	3 (13.6)	0.060
Periampullary diverticula	19 (14.8)	1 (%4.5)	0.308
History of gastric operation	5 (3.9)	0	0.446
Impacted stone	14 (%10.9)	3 (%13.6)	0.375
Stricture distal to the stone	27 (%21)	6 (%27.2)	0.379
Number of ERCP sessions	3.34 ± 1.57 (1–10)	3.36 ± 2 (1–9)	0.558
Total follow-up (months)	5 ± 7.5 (0.75–39.75)	5.1 ± 7.5 (1–61)	0.780
Bile duct size in first session (mm)	14.8 ± 3.8 (7–26)	20.7 ± 7.3 (11–33)	0.006
Bile duct size in last session (mm)	13.1 ± 3.68 (7–25)	19.3 ± 7 (11–29)	<0.001
Number of patients with reduced CBD diameter *n* (%)	106 (82.8)	7 (31.8)	0.001
Number of patients with more than 3 stones at initial session; *n* (%)	75 (58.5)	13 (46.4)	0.931
Maximum stone size in first session (mm)	15.8 ± 5.5 (12–30)	27.7 ± 10.3 (24–44)	<0.001
Maximum stone size in last session (mm)	13.3 ± 4.1 (6–24)	26.1 ± 7.5 (22–41)	<0.001
Number of patients with reduced diameter of the largest stone; *n* (%)	97 (75.7)	16 (57.1)	0.413
Additional technique; *n* (%)			
Mechanical lithotripsy	25 (19.5)	2 (7.1)	0.252
Balloon sphincteroplasty	6 (4.6)	1 (3.5)	0.591

Values are presented as number (%), mean ± SD, or median (range).

ALT: alanine aminotransferase; AST: aspartate aminotransferase; CBD: common bile duct; ERCP: endoscopic retrograde cholangiopancreatography; GGT: gamma glutamyl transferase; ALP: alkaline phosphatase.

**Table 3:** Multivariate analysis for factors affecting endoscopic success.

Variable	OR	%95 CI	*p *value
Age	0.963	(0.921–1.006)	0.089
Previous cholecystectomy	1.006	(0.997–1.015)	0.181
Periampullary diverticula	1.040	(0.315–1.780)	0.774
Bile duct size in first session	0.780	(0.681–0.893)	0.001
Maximum stone size in first session	0.808	(0.726–0.898)	0.001

**Table 4:** Published cases of papillary balloon dilation, mechanical lithotripsy, and laser lithotripsy for difficult bile duct stones.

Ref.	Treatment	Total number of patients	Stone size (mm)	Success rate (%)
Schneider et al. [15]	ML	209	4–80	87.6
Hintze et al. [16]	ML	84	>15	98.4
Garg et al. [17]	ML	87	>15	79.0
Cipolletta et al. [18]	ML	162	9–50	84
Rosa et al. [20]	ESPBD	68	12–30	95.6
Stefanidis et al. [21]	ESPBD	45	12–20	97.7
Minami et al. [22]	ESPBD	88	>12	99
Bang et al. [23]	ESPBD	22	5–25	72.7
Sauer et al. [27]	LL	20	11–35	90
Hochberger et al. [28]	LL	60	>10	87
Lee et al. [29]	LL	10	16–23	90
Maydeo et al. [30]	LL	60	15–25	100

ML: mechanical lithotripsy; ESLBD: endoscopic sphincterotomy and papillary balloon dilation; LL: laser lithotripsy.
